# The Medical Profession in Social Estimation

**Published:** 1909-12-25

**Authors:** 


### DOCTOR AND LAYMAN.

### THE MEDICAL PROFESSION IN SOCIAL ESTIMATION.

In a recent annotation in this journal it was pointed out that on the whole doctors are thought most of, and paid most attention to, in countries most democratic in sentiment; even if their form of government happened to be monarchical. The comment was added that the fact is encouraging to the British medical profession in view of present-day political tendencies. Much incitement to thought, indeed, both retrospective and anticipatory, may be found in the subject thus defined. It cannot be denied that in the past, whether from faults of its own or not, the position taken by medicine in the regard of the community has not been such as to gratify its votaries very highly. What certain of our political friends, who are so much in evidence just now, would call persistent vestiges of feudality—or some such facile and high-sounding phrase—does not seem to tend to an unrestricted healthy development of medicine and its rise to its rightful place in a nation's councils. The trend of aristocratic and plutocratic sentiment both is, by virtue of the nature of each, somewhat adverse to these objects; for neither privilege nor first-rate business instinct can afford to concede too much to intellect—government by which is not at present regarded by judges of the matter as likely to prove an unmixed blessing. And intellect is requisite to eminence in medicine, although not so much so as many people think. Straws may show the direction of this wind of favour. The position, for instance, of European court physicians at various functions (though not in cases of Imperial or Royal illness) is quite a subordinate one. There is a story, too, which, even if apocryphal, still remains a good allegory, of an aged peer who, at the time of Lord Lister's entering the House of Lords, protested in horror against making a peer of a man who, if he, the speaker, entered his consulting-room complaining of certain symptoms, and paid him two guineas, would have to make a rather unpleasant physical examination of his, the speaker's, venerable person. Not incongruous with this fable is the picture of the medical man in his social relations drawn by some popular novelists. In an early book of Mr. Anthony Hope's, there is a note of almost personal solicitude in the passage where he invites the reader to remark the goodness of the squire's lady in making friends with the village doctor's wife. And Mr. Stanley Weyman, in a mild (and also juvenile) work which makes one recall *Barchester Towers* with regret, has drawn his doctor despicable, futile, and bad, and his clergy just the opposite. Even Mr. Henry James, whose minute view of a social scene, although not wholly uncoloured by prepossessions in favour of gentility, deserves nevertheless great respect, has dropped a sneer or two. In spite of all the glossing over which, in these times of glib soft speaking, unseemly facts are sure to come in for, it is fairly evident that in the past, at any rate, there have been quarters from which the medical profession has had to encounter prejudice.

The innovating ideas of Huxley went down fairly well when presented in the form of articles in the reviews signed by eminent people, but when it came to their being preached or practised in every-day life by ardent, less distinguished, less fortunately-placed, and possibly rather indiscreet and tactless partisans, they aroused the bitterest opposition, quite irrespective of the question of their intrinsic merit. And then medicine has long taken the ancient place of the Church as a profession by means of which lowly-born but talented individuals may rise from the masses, at no risk of losing their bread and butter even if they make a comparative failure of their ambitious attempt. A nation characterised as ours has been by Thackeray, a man of talent unbiassed by humble extraction, will not have much respect to spare for a calling which does not require of its followers considerable initial resources and special influence, qualifications necessary in the case of diplomacy or the combatant services. That this does not hold good of countries where, probably, things of the mind are more esteemed, was curiously illustrated a few years ago by a legal case on the Continent, in the course of which it came out that an Italian nobleman, jealous of the academic society which absorbed so much of his wife's attention and admiration, set to work to acquire some medical knowledge in order to polish himself. Truth is stranger than fiction, of course; but it is to be feared that the reviewers would make inconveniently merry at the expense of any author who should depict such a situation in a novel of English life.

Of more importance, most will think, than these reflections, in which, maybe, reversing the usual course of memory, ills have figured too largely, and benefits have been lost sight of, is the question of the social estimation of the medical profession in its bearing on that democratic control of institutions and affairs which is becoming more manifest in this country almost daily. There is no doubt it is a burning one. Whether, as some say, Snug the joiner and Bottom the weaver are going to prove the worst of new masters, or whether, on another extreme view, pride of birth and purse will no longer overshadow the practice of the medical profession and modify the diffusion of the teachings of medical science and hygiene. It is in the provinces that most of the friction attendant upon this change, as upon all sudden ones, is likely to arise, for Metropolitan institutions for the benefit of the sick are supported largely by moneyed individual subscribers. But when, in large manufacturing towns and cities, working men governors of hospitals are common, as, on every principle of fair representation they must and should be, and where municipal authorities contain a large "labour" element, it is not hard to foresee that situations rather delicate in the view of those who are their skilled medical servants —skilled, certainly, but still servants—not delicate at all to the matter-of-fact lower middle-class leader, may crop up pretty frequently. Personal feeling, so powerful in all human relations, is likely to be especially powerful here; and so the real estimation in which medical men are held by the lower social classes is a matter which it becomes very important to gauge correctly. Are they likely to think him dictatorial, self-interested and class-interested, already over-paid; or will they accept his guidance in the solution of certain pressing medico-social problems, even including one or two of the evils which oppress his own lot?

Unfortunately a good many members of the profession seem to be of the former opinion. Whoever has talked on this subject with the medical men of a large provincial city must have noticed symptoms of class feeling. Some, indeed, profess themselves ready to condone downright reaction and illiberality if only the process of levelling up to themselves may be checked; and amongst these are able clinicians. The tact which is happily a common attribute of middle-aged Englishmen, seems too often wanting when this particular subject comes up for discussion—as it does so often—and a rather unaccustomed bitterness is apt to appear. Tales are told of artisans so impressed with the dignity conferred upon them by their newly won civic titles that they use them even when visiting the private houses of their customers in the way of business; or of a committee of working men censuring the medical officer of an institution whom they find playing lawn tennis or croquet on a summer afternoon (according to their way of looking at things, a flagrant case of idleness of an employé during working hours); or of collier patients inquiring of a general practitioner's well-qualified assistant whether or not he is "Brown's man." A little cross-examination will usually, however, reveal the fact that no real animus against medical men, or against the social class from which they are commonly drawn, underlies these *gaucheries*. The respect which an able and hard-working medical man who is fond of his profession commands from the laity is nowhere stronger—nowhere so strong in fact—as amongst the lower orders. They will often repose in him a loyal faith, which in return touches him deeply, and gives a peculiar poignancy to the regret he feels over failure in his endeavours for their physical welfare. It has yet to be found that they or their leaders entertain any jealousy of the initiative power that should appertain to medical as to other experts. They have few vested interests to make obstructionists and obscurantists of them. In their cases, the due deference and respect of a man ignorant of a difficult subject for one well versed in it is not vitiated by any factitious social pride. The practitioner of medicine has no traditional ascendency over them, a thing which it may be right or wrong for them *per se* to resent, but which as an actual matter of fact they do resent all the same. The rise of the medical profession to an undeniable place of influence in the practical affairs of the community, secured as it has been by the work of the last few decades in demography and bacteriology, does not ante-date by much the rise of democratic political power. And, as already mentioned, since the passing of the Education Act it has become increasingly common for talented youths "of the people" to seek a career for themselves in medicine. In short, no surprise need be felt at the observation that nowhere is the smile of greeting, which, it is pleasant to think, every practitioner on his rounds must be extremely familiar with—nowhere is this expression of good feeling so genuine as amongst the common people. It is to be hoped, then, not merely for the sake of putting a good face upon the inevitable, but from higher and better motives, that medical men will seek for points of sympathy with those classes of the laity that seem to be entering upon a period of responsibility, and allow those feelings to prevail which are not only customary at this season of the year, but also necessary to the sweetening of human relations and the practical furtherance of progress.

